# Improving osteoinduction and osteogenesis of Ti6Al4V alloy porous scaffold by regulating the pore structure

**DOI:** 10.3389/fchem.2023.1190630

**Published:** 2023-05-17

**Authors:** Chao Wang, Jie Wu, Leyi Liu, Duoling Xu, Yuanbo Liu, Shujun Li, Wentao Hou, Jian Wang, Xun Chen, Liyuan Sheng, Huancai Lin, Dongsheng Yu

**Affiliations:** ^1^ Hospital of Stomatology, Guanghua School of Stomatology, Sun Yat-sen University, Guangzhou, China; ^2^ Guangdong Provincial Key Laboratory of Stomatology, Sun Yat-sen University, Guangzhou, China; ^3^ Institute of Metal Research, Chinese Academy of Sciences, Shenyang, China; ^4^ Shenzhen Institute, Peking University, Shenzhen, China

**Keywords:** porous scaffold, Ti6Al4V alloy, pore structure, osteogenesis, bone defect repairing

## Abstract

Titanium alloy scaffolds with a porous structure have attracted much attention in bone defect repair. However, which pore structure is more beneficial to bone defect repair is controversial. In the present research, the Ti6Al4V alloy porous scaffolds with gradient pore sizes were designed and fabricated. The microstructure characterization, tests of mechanical properties, and *in vitro* and *in vivo* experiments have been performed to systematically evaluate the effect of pore size on osteoinduction and osteogenesis. The results revealed that the contact angle with water, compressive strength, and elastic modulus of the Ti6Al4V alloy porous scaffolds decreased gradually with the increase of pore size. However, there were obvious drops when the pore size of the porous scaffold was around 600 μm. As the pore size increased, the proliferation and integrin β1 of RAW 264.7 macrophages seeded on Ti6Al4V alloy porous scaffolds increased at first, reaching a maximum value at a pore size of around 600 μm, and then decreased subsequently. The proliferation, integrin β1, and osteogenic gene-related expressions of Bone marrow mesenchymal stem cells (BMSCs) seeded on Ti6Al4V alloy porous scaffolds with different pore sizes all exhibited similar variations which rose with increased pore size firstly, obtaining the maximum value at pore size about 600 μm, and then declined. The *in vivo* experiments confirmed the *in vitro* results, and the Ti6Al4V alloy porous scaffold with a pore size of 600 μm possessed the better capability to induce new bone formation. Therefore, for the design of Ti6Al4V alloy with a regular porous scaffold, the surface morphology, porosity, strength, and elastic modulus should be considered systematically, which would determine the capability of osteoinduction and osteogenesis.

## 1 Introduction

Repair of bone defects in the oral maxillofacial region caused by severe trauma, malignant tumors, and innate diseases remains a crucial clinical issue (Ruales-Carrera et al., 2019). Though related research has been performed in the past decades, bone grafting is still the gold standard for bone defect repair (Biggemann et al., 2018). However, donor site morbidity, limited tissue availability, and additional surgical pain have greatly constrained its clinical application (Chen et al., 2022). Fortunately, the recent progress in bone tissue engineering by scaffold-inducing osteogenesis have provided a promising strategy for the reconstruction of bone defects (Wang et al., 2022). Due to the requirement of mechanical properties and biocompatibility on the scaffold, the titanium alloy scaffold with a porous structure has attracted much attention in the field of bone defect repair (Zhang et al., 2018; Tsai et al., 2021). Its porous structure could benefit the transport of nutrients, and the inward growth and differentiation of bone cells (Chen et al., 2022). In addition, the reduced elastic modulus contributes to interface compatibility and promotes bone stability (Wang et al., 2022). However, the titanium alloy scaffold still has a higher relative elastic modulus, compared with the bone tissue. The “stress shielding” effect would result in poor bone bonding with scaffolds, even aseptic loosening. The recent research revealed that the mechanical properties of the porous scaffold were closely related to its pore size and porosity (Lv et al., 2022). However, considering the biocompatibility requirement, it is necessary to take a thorough investigation on the influence of titanium alloy porous scaffold structure.

Recently, many researchers (Li et al., 2012; Zhang et al., 2018; Tsai et al., 2021; Lv et al., 2022) have demonstrated that the elastic modulus of titanium alloy porous scaffolds can be reduced by increasing pore size and porosity. The study on Ti6Al4V porous scaffold revealed that the small increase of pore size from 0.5 mm to 0.65 mm could decrease its elastic modulus to 14.5 GPa which is close to that of adult cortical bone (17–20 GPa) and almost half of the original value (Li et al., 2012). However, such an elastic modulus value is still much higher than that of cancellous bone (0.76–4 GPa). Heinl et al. decreased the elastic modulus of the Ti6Al4V porous scaffold from 12.9 GPa to 1.6 GPa by increasing the pore diameter and porosity from 0.45 mm to 59.5% to 1.23 mm and 81.1%, respectively (Heinl et al., 2008). Though the increased pore size could reduce the elastic modulus of the titanium alloy porous scaffold, the strength is decreased simultaneously (Karageorgiou and Kaplan, 2005). The compressive strength of the cortical bone and cancellous bone is about 180–210 MPa and 2–5 MPa, respectively (Barth et al., 2011). How to balance the strength and elastic modulus of the scaffold is still an urgent research issue, which influences its bone integration behavior and safety.

Generally, the bone integration ability of titanium alloy porous scaffolds is determined by its bone conductivity which is mainly affected by the porosity and the pore size. When the pore size of the porous scaffold is about 500 μm, the proliferation and differentiation of osteoblasts on it are faster than those on the scaffold with a pore size less than 200 μm (Jamaloei and Kharrat, 2011). Kapa et al. reported that the porous scaffold with an average pore size of 500 μm exhibited extensive cell coverage, which benefited the mesenchymal stem cells (MSCs) differentiating into the bone lineage and strong ability of osseointegration (Kapat et al., 2017). The research indicated that the porous scaffolds with the pore size of 300–1,000 μm would be much beneficial to the adhesion, proliferation, and differentiation of osteoblasts (Frosch et al., 2002). However, such a size range is really so big that it is difficult for the implant design. Especially for the specific bone defect repairing, the strength, elastic modulus and osseointegration ability of the scaffold should be considered systematically. Our previous research revealed that porous titanium alloy scaffolds with large pore sizes could promote the formation of blood vessels and the growth of new bone (Wang et al., 2020; Wang et al., 2021). However, it is still a question to explore what environmental factors and which cell types are mainly involved during the osteoblastic process. In fact, the migration of immune cells around the scaffold setup an immune microenvironment that is jointly participated by macrophages, osteoblasts, and other cells, and moreover its regulation determines the fate of osteoblast differentiation and the results of bone healing (Sridharan et al., 2015; Miron and Bosshardt, 2016). Therefore, the systematic investigation on the titanium alloy porous scaffold should be performed.

Based on the previous studies, the Ti6Al4V alloy porous scaffolds with a regular pore shape from 300 um to 1,000 μm were designed and fabricated by electron beam melting (EBM) technology. Their macrostructure, mechanical properties, and *in vitro* and *in vivo* experiments were performed to explore the optimal pore size and porosity that are beneficial to osteogenic differentiation. In addition, the mechanism of porous scaffolds promoting osteoblast proliferation and differentiation in the immune microenvironment was discussed for better understanding the influence of porous structure.

## 2 Experimental details

### 2.1 Sample preparation and characterization

The porous scaffolds with regular cubic pore and sizes of 300 μm, 400 μm, 500 μm, 600 μm, 700 μm, 800 μm, 900 μm, and 1,000 μm were designed by Medica software (Autodesk, CA) and named as P300, P400, P500, P600, P700, P800, P900, and P1000, respectively. The porous scaffolds were prepared by EBM printing (ARCAM A1, Gothenburg, Sweden) from Ti6Al4V alloy powders for the following characterization and test. One kind of porous scaffold with a 10 mm diameter and 2 mm thickness is prepared for *in vitro* cell experiment, while the other kind of porous scaffold with 5 mm diameter and 8 mm height is prepared for *in vivo* animal experiment. The design and printing of various scaffolds were completed at the Institute of Metal Research, Chinese Academy of Sciences. All prepared porous scaffolds were ultrasonically cleaned in acetone, ethanol, and deionized water for 60 min, respectively. The cleaning process was repeated three times, followed by autoclaving at 121°C for 60 min. After then the pre-treated scaffolds were placed in a constant temperature oven at 50°C. The porous scaffolds would be subjected to UV irradiation before the *in vitro* and *in vivo* experiments.

The surface morphology of the porous scaffold was observed using a scanning electron microscope (SEM; JSM 6700, JEOL, Japan) after surface gold spraying. The test method and test results of the elemental composition of the porous scaffolds were consistent with those in our published article (Wang et al., 2021). A contact angle measuring instrument (Kruss, DSA30, Germany) was used to test the surface wettability of the porous scaffolds. Water from the syringe on the surface tension tester was dropped onto the sample surface, and images were taken after stabilization. The analysis was also performed by the tester’s own software. In order to evaluate the mechanical properties of porous scaffolds, compression testing was performed on the electronic universal testing machine (INSTRON 5582) with a cross-head speed of 1.5 mm/min. Three porous scaffolds with the same pore size were tested to obtain one group of data. The compressive properties, including the quasi-elastic modulus and compressive strength, were calculated from the stress-strain curve.

### 2.2 *In vitro* cell experiments

#### 2.2.1 Culture of RAW 264.7 and bone marrow mesenchymal stem cells (BMSCs)

The mouse RAW 264.7 cell line (ATCC^®^ TIB-71TM) was added to the DMEM medium with 10% fetal bovine serum (FBS) and 1% penicillin/streptomycin incubated at 37°C in a humidified incubator with 5% CO_2_. When cell fusion reaches 80% or more, cells are counted by trypsin digestion and inoculated on the scaffold at a density of 5×10^4^cells/cm^2^.

The procedures for preparation of bone marrow mesenchymal stem cells (BMSCs) were modified by the literature (Truong et al., 2019). The SD rats are larger than mice, having greater bone mass and more bone marrow content, so more mesenchymal stem cells can be extracted. So, the SD rats were selected for the preparation of BMSCs.SD rats of about 10 days old were selected and sacrificed by cervical dislocation. The metaphysis of mouse femurs and tibias was cut off to expose the medullary cavity so that the marrow plug can be flushed out of the cut end of the bone with complete medium (DMEM-F12 medium containing 10% fetal bovine serum). After BMSCs were collected from bone marrow, the red blood cells should be lysed before culturing in the medium. The cells were cultured in DMEM-F12 supplemented with 10% fetal bovine serum (FBS, Hyclone), 100 IU/mL penicillin, 100 IU/streptomycin, and 4 ng/mL fibroblast growth factor basic (FGF, Peprotech). Cells of passages 3–5 were used to complete subsequent experiments.

#### 2.2.2 Cell viability and proliferation assay of RAW 264.7

Cell viability was evaluated by Live/Dead Cell Kit (Solarbio, China). On 1, 3 days, scaffolds were washed with PBS and then transferred to a new 24-well plate. After culture in 500 μL DMEM with 1-mL quantity of Hanks’ Balanced Salt Solution (HBSS) diluted solution A (calcein AM) for 25 min. Then, a 1-mL quantity of diluted B solution was placed on the scaffolds and incubated in the dark for 3 min. The cells were then gently washed twice with phosphate-buffered saline (PBS). The cell viability of RAW 264.7 on scaffolds was observed by laser confocal microscopy (LSM780, Zeiss, Germany).

CCK-8 kit (Dojindo, Japan) was used to determine the cell proliferation ability. The scaffold was seeded with RAW 264.7 in a 24-well plate. On the 1st, 3rd, 5th, and 7th days, 10% CCK-8 determination solution was added to the medium and incubated for 2 h. The optical density of the wells was read on a microplate reader for cell proliferation assay.

#### 2.2.3 Immunofluorescent staining of RAW 264.7

The porous scaffolds with different pore sizes were placed on the well bottom of the 24-well plate. Mouse RAW264.7 cells were digested, counted, and evenly inoculated on the surface of scaffolds with 5×10^4^ cells/mL and 1 mL/well. The cells were terminated after the 3rd day of cell culture and fixed with 4% paraformaldehyde solution at room temperature for 10 min. The membrane was broken by soaking with 0.5% Triton-X (diluted with PBS) for 10 min, then closed with 5% BSA milk closure solution for 1 h at room temperature, followed by washing with PBS for one time. Integrin β1 primary antibody (Affinity) diluted at 1:200 was added to the wells and left overnight at 4°C. Add secondary antibody (Affinity) diluted 1:200 to the wells and incubate for 1 h at room temperature. The FITC-labeled Cyclic Peptide powders were dissolved in 1.5 mL of methanol solution and configured as a storage solution (20×). Preparation of working solution: 5 μL of the master batch of FITC was added to 95 μL of PBS to make a working solution. The 200 μL of the working solution was added to the surface of the specimen per well, and the incubation is 30 min at room temperature, avoiding light.

#### 2.2.4 Configuration of macrophage conditioned medium and BMSCs osteoblastic differentiation on scaffolds

The supernatant ofRAW 264.7 cultured for 24 h was collected and prepared into the conditioned medium with DMEM in a ratio of 1:1. The cell viability test was performed as in 2.2.2. The scaffolds were placed on the well bottom of the 24-well plates. BMSCs cells were digested, counted, and diluted at a cell concentration of 5×10^4^ cells/mL. BMSCs were uniformly inoculated onto the surface of the scaffolds with different pore sizes at 1 mL/well. Unadhered cells were washed off with PBS, and then 2.5% glutaraldehyde was added overnight at 4°C. The scaffolds were then dehydrated with different concentrations of ethanol (30%, 50%, 70%, 80%, 90%, and 100%) in a gradient of 10 min each time. After then, the scaffolds were dried and treated with gold spray. The 10 KV voltage was set in SEM to observe the cell morphology of BMSCs on the scaffolds.

#### 2.2.5 Immunofluorescent staining of bone mesenchymal stem cells (BMSCs) and BMSCs on scaffolds

The immunofluorescence staining of integrin β1 on BMSCs and scaffolds for BMSCs was performed as in 2.2.3.

#### 2.2.6 Expression of osteogenic genes

To assess the expression of osteogenic-related genes in BMSCs, cells were cultured on porous scaffolds for 7 and 14 days, respectively. We extracted total RNA using the TRIzol method and converted the RNA into cDNA by Prime Script RT Master Mix (Takara), which was then amplified by PCR to generate products corresponding to the mRNA-encoded human gene products listed in Table 1.

**TABLE 1:** Primers used for quantitative RT-PCR analysis.

Gene ALP	Forward primer sequence(5'-3') AGC​GAC​ACG​GAC​AAG​AAG​C	Reverse primer sequence(5'-3') GGCAAAGACCGCCACATC
Ruma	GCG​GTG​CAA​ACT​TTC​TCC​AG	TCA​CTG​CAC​TGA​AGA​GGC​TG
OCN OPN	GAG​GGC​AGT​AAG​GTG​GTG​AA GACAGCAACGGGAAGACC	CGT​CCT​GGA​AGC​CAA​TGT​G CAGGCTGGCTTTGGAACT
VEGF	CAA​ACC​TCA​CCA​AAG​CCA​GC	CGC​CTT​GGC​TTG​TCA​CAT​TTT​T
KDR	GAC​AAC​CAG​ACG​GAC​AGT​GG	TGG​GCA​CCA​TTC​CAC​CAA​AA
GAPDH	AGT​GCC​AGC​CTC​GTC​TCA​TA	GAT​GGT​GAT​GGG​TTT​CCC​GT

### 2.3 *In vivo* animal experiments

**FIGURE 1:**
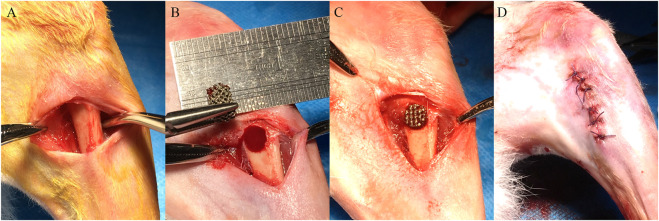
The surgical procedure of porous scaffold implantation in the bone defect. **(A)** Skin preparation in the operative area to expose the femur. **(B)** Preparation of the bone defect step by step. **(C)** Implantation of porous scaffolds with different pore sizes. **(D)** Suture of the wound.

A cohort of 28 adult male New Zealand rabbits weighing 2.5–3.0 kg were selected for *in vivo* experiments. All animal experiments followed relevant laws and regulations, and this experiment was approved by the Laboratory Animal Ethics Committee of Sun Yat-sen University (Approval number: SYSU-IACUC-2019–000169). After injecting 3% sodium pentobarbital (30 mg/kg) into a New Zealand rabbit’s ear vein, a longitudinal incision was made in the skin of the proximal femur to expose the bone surface. A model of the bone defect with a diameter of 5 mm and a depth of 8 mm was made by a drill at 500 r/min using a step-by-step hole preparation method, and porous scaffolds with different pore sizes were implanted sequentially. The wound was then tightly sutured in layers. The surgical procedure is shown in Figure 1.

#### 2.3.1 Micro-CT scanning analysis

At the 12th week, the porous scaffold implanted site was scanned with Micro-CT (Nanovoxel 3000d, Sanying Precision, China) to observe the osteogenesis of the porous scaffold *in vivo*. Four samples were taken from each group for Micro-CT scanning. Under 120 kV voltage, 110 uA current, and 10 µm accuracy, the implant and its surrounding bone tissue were scanned. Mimic 15.0 software was used for the 3D reconstruction of Micro-CT image data. The above Micro-CT scanning and data analysis were completed at Guangdong University of Technology.

#### 2.3.2 Tissue sections analysis

The rabbits with porous scaffold implanted in the femur were killed at 12 weeks to obtain the tissue specimens. The tissue with implanted porous scaffold was excised by an ultra-hard blade. The excised tissues were fixed in 4% buffered formaldehyde (Sigma) and decalcified with 17% ethylenediaminetetraacetic acid (EDTA, Sigma) followed by embedding in paraffin. Then the tissue was stained with hematoxylin-eosin (H&E) and modified Van Gieson staining for histological analysis.

### 2.4 Statistical analysis

Data were calculated as the mean ± standard deviation. Each group of data was obtained using three independent experiments. Differences between multiple experimental groups were statistically analyzed using a one-way analysis of variance and Tukey’s multiple comparison test. GraphPad Prism v.8.0 was utilized for all statistical analyses. A significant difference was indicated by *p* < 0.05.

## 3 Results

### 3.1 Morphology and properties

**FIGURE 2:**
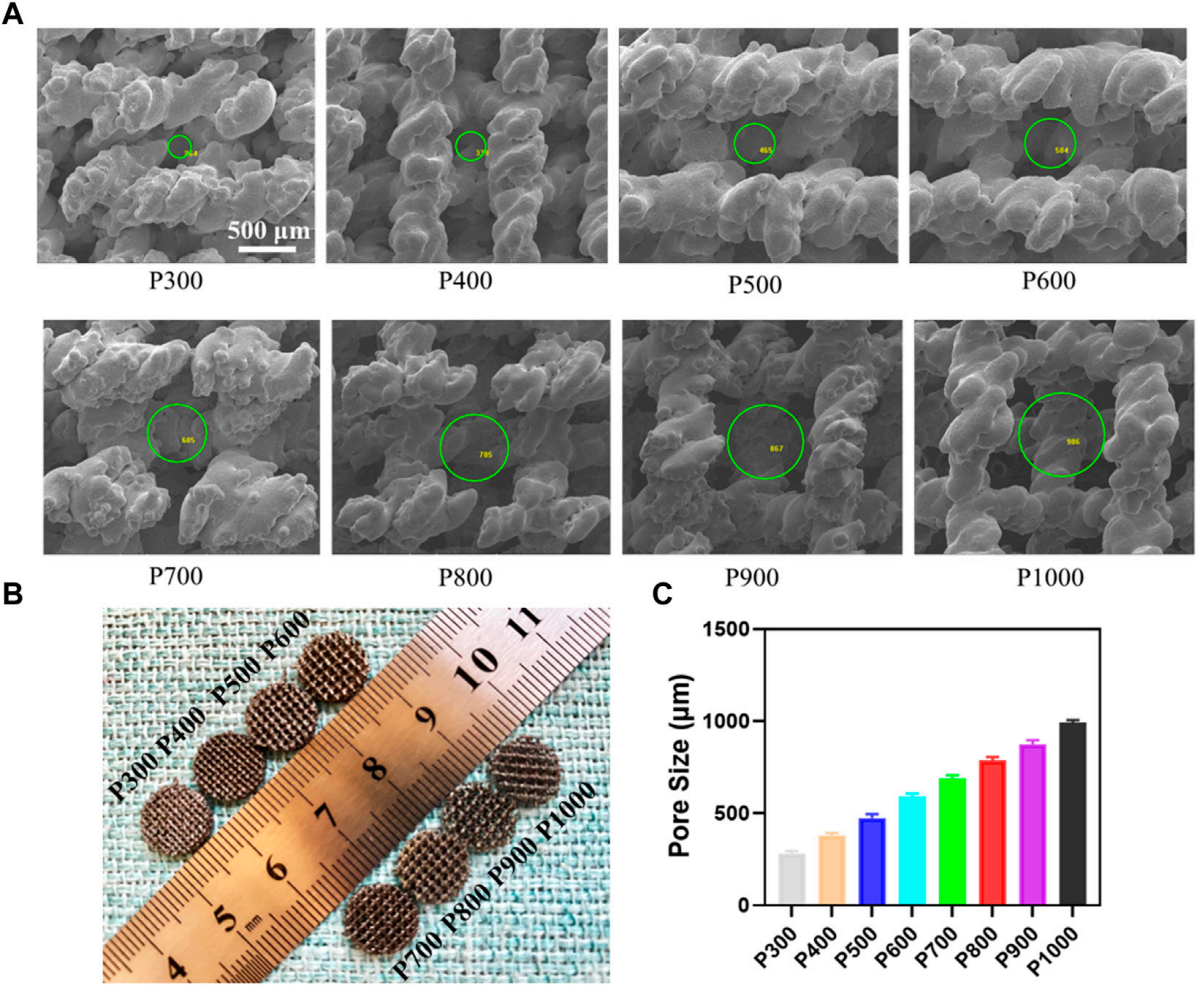
Morphology of the Ti6Al4V alloy porous scaffolds and their pore size analysis. **(A)** Morphology of porous structure by SEM and pore size measured using Image-J software. **(B)** The macrograph of the porous scaffolds with different pore sizes. **(C)** Statistical analyses of the pore size in different porous scaffolds.

The as-prepared Ti6Al4V alloy porous scaffolds still had the features of solidified powders, as shown in Figure 2A. The size of the molten pool could influence the boundary of the pore structure, which resulted in the thread feature in a linear frame. Especially for the porous scaffold with a small pore size, the morphology of the pore was affected obviously, due to the definite frame size. With the increase of pore size, the pore changed gradually into the regular cubicform. The macrographic observation on the Ti6Al4V alloy porous scaffolds demonstrated the increased pore size enhanced the permeability, which would be beneficial to the transfer of nutrients, as shown in Figure 2B. Based on the SEM observations, the statistical analyses on the pore size by Image-J software showed that the pore size increased from about 280 μm to 990 μm, as shown in Figure 2C. In addition, the pore size almost kept the linearly increasing with the same slope.

**FIGURE 3:**
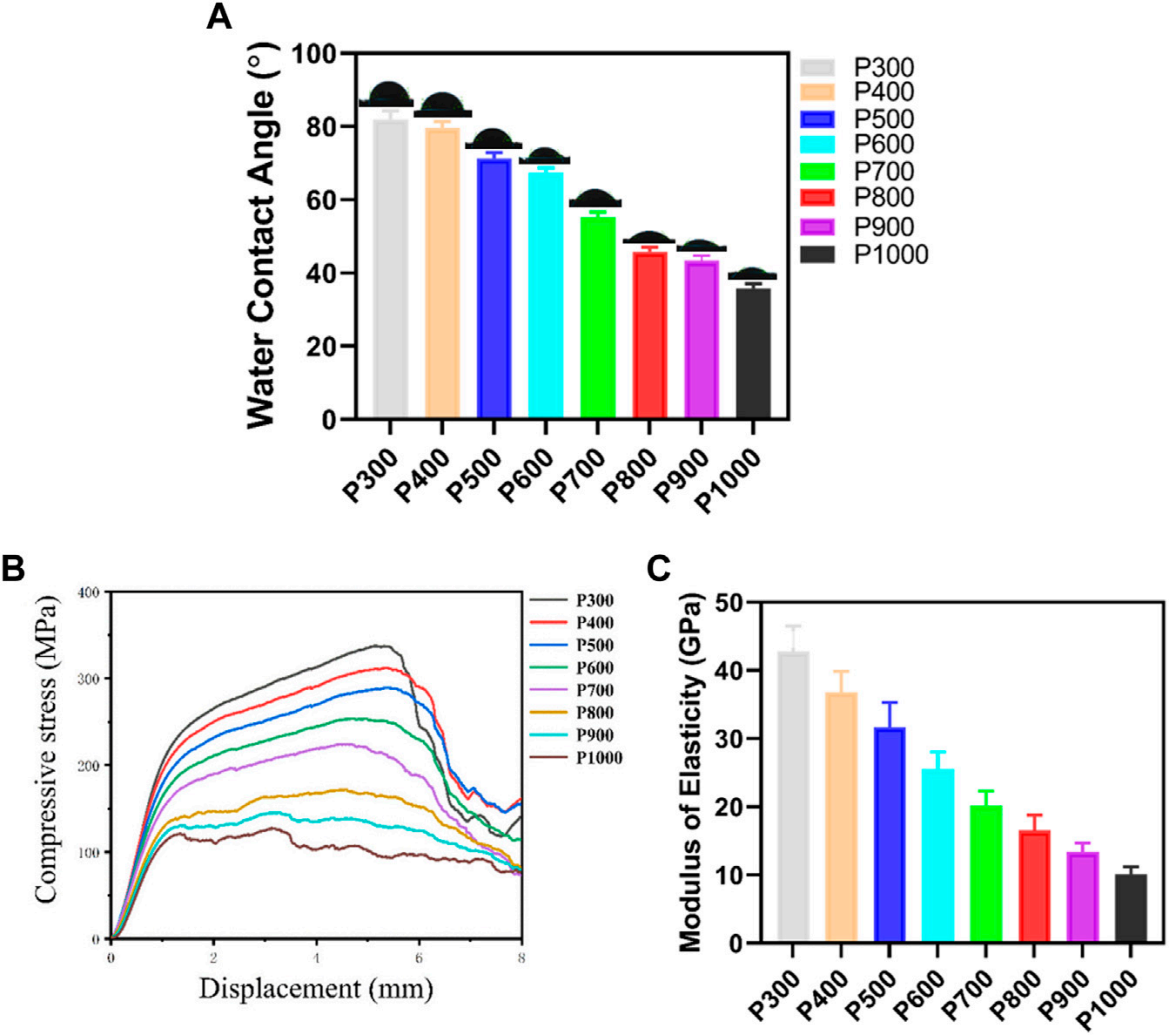
Physical and mechanical properties of the Ti6Al4V alloy porous scaffolds with different pore sizes. **(A)** Variation of contact angle of water on porous scaffolds with different pore sizes. **(B)** Compressive stress-displacement curves of porous scaffolds with different pore sizes. **(C)** Elastic modulus of porous scaffolds with different pore sizes.

Due to the solidified surface and pore size, the Ti6Al4V alloy porous scaffolds would demonstrate different physical and mechanical properties. As shown in Figure 3A, the contact angle of water on the porous scaffold decreased gradually with the increase of pore size. When the pore size was smaller than 400 μm, the decrease in contact angle was small, but the contact angle had a little drop between the pore size of 400 μm and 500 μm. What was interesting was that the contact angle had an obvious drop, when the pore size was between 600 μm and 800 μm. When the pore size decreased further, the contact angle decreased gradually and had a little drop at a pore size of 1,000 μm. The P300 porous scaffold had the largest contact angle with water of 81.99 ± 2.30° and the P1000 porous scaffold had the smallest one of 35.82 ± 1.22°. The compressive tests revealed that the porous scaffolds exhibited the typical yield and plastic deformation behavior, as shown in Figure 3B. The yield strength and compressive strength of the porous scaffold increased with the pore size. It was interesting that the strength of the porous scaffold had an obvious jump between pore size of 700 μm and 800 μm. At the last stage of compression, the porous scaffolds almost demonstrated a similar strength degeneration tendency except for the P300 porous scaffold with more rapid degeneration. The P300 porous scaffold had the highest yield strength of 185.86 ± 5.16 MPa and compressive strength of 337.49 ± 11.24 MPa. The P1000 porous scaffold had the lowest yield strength of 90.45 ± 3.51 MPa and compressive strength of 127.41 ± 6.28 MPa. Based on the compressive curves, the Young’s modulus of elasticity of each porous scaffold was calculated and demonstrated in Figure 3C. Clearly, the elastic modulus of the porous scaffold decreased gradually with the increasing of pore size, and the decreasing rate with pore size showed almost the same slope. The P300 porous scaffold had the highest elastic modulus of 42.71 ± 3.82 GPa, while the P1000 porous scaffold had the lowest one of 10.12 ± 1.12 GPa.

### 3.2 *In vitro* cell experiments

#### 3.2.1 Cell viability and proliferation assay of RAW 264.7

**FIGURE 4:**
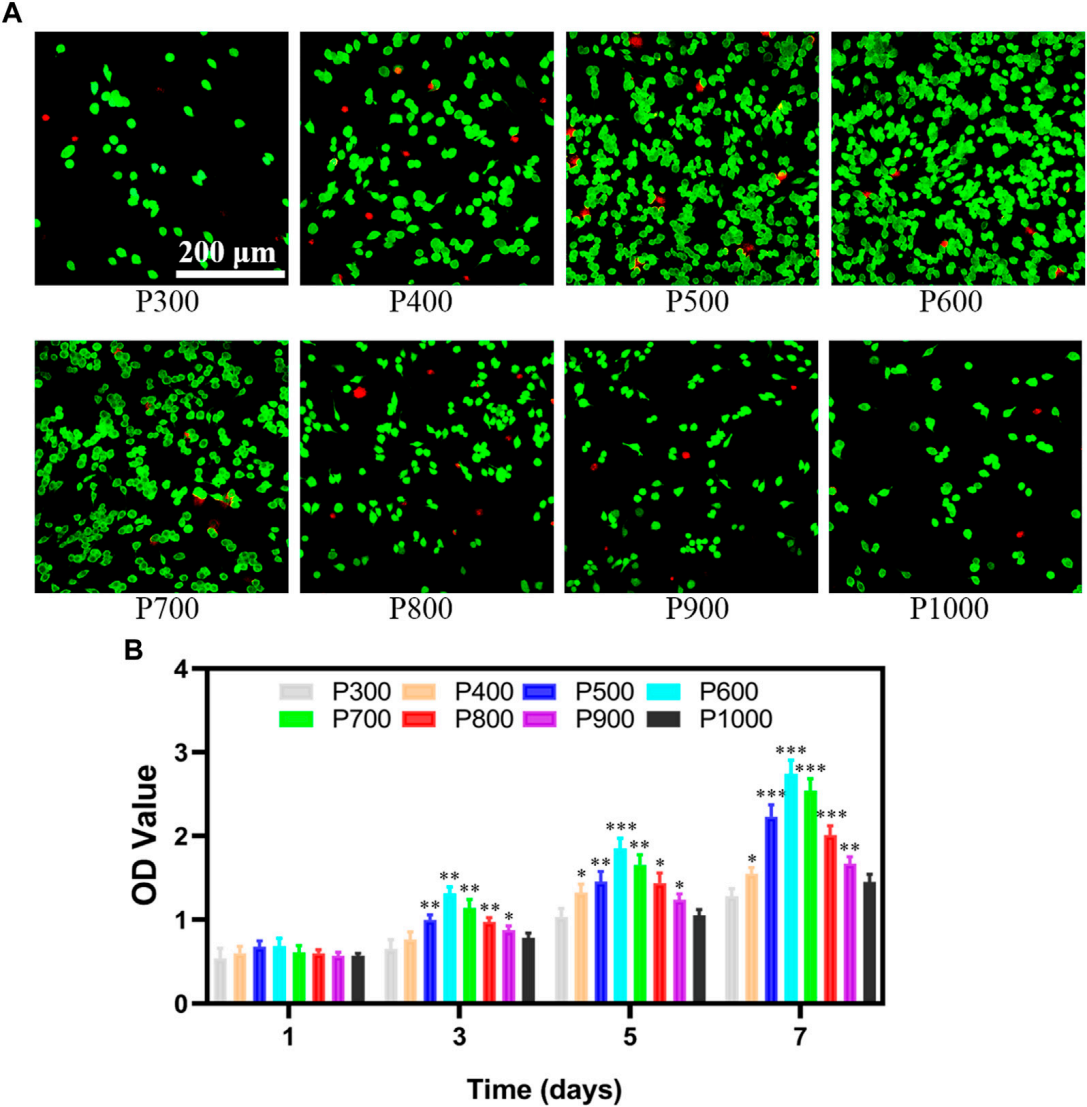
The viability and proliferation of macrophages on porous scaffolds with different pore sizes. **(A)** Detection of macrophage viability on porous scaffolds with different pore sizes. **(B)** CCK8 test results of macrophage proliferation ability on porous scaffolds with different pore sizes (*p < 0.05, **p < 0.01, and ***p < 0.001).

The macrophages are seeded on porous scaffolds and cultured in a high-glucose MEMD medium for 1 day. The macrophages adhered to the scaffold are washed off with PBS, and the detached cells are then placed in a new 24-well plate and cultured in high-glucose MEMD medium for 1 day further. As shown in Figure 4A, most of the macrophages were in a fried egg-like M1 polarized state, with mostly live cells (fluorescence staining in green) and a few dead cells (fluorescence staining in red) interspersed. It can be found that the number of macrophages exhibited an obvious change on porous scaffolds with pore size increasing. When the pore size is smaller than 600 μm, the number of macrophage adhesion increased gradually with pore size increasing (P300, P400, P500, and P600). However, the amount of macrophage adhesion would decrease rapidly with pore size increasing further (P700, P800, P900, and P1000). Correspondingly, the proportion of dead macrophages to the total at first decreased and then increased. The proliferation ability of macrophages on scaffolds with different pore sizes was measured by CCK-8 kit and shown in Figure 4B. On the first day, the macrophages seeded with porous scaffolds with different pore sizes almost demonstrated no significant difference in the OD value ranged from 0.54–0.68. The OD values of the macrophages seeded on the P500 and P600 porous scaffolds had slightly higher values. As time increased, the OD values of macrophages on porous scaffolds all exhibited an increasing tendency, but the ratio of increase was different. The OD values of macrophages on porous scaffolds on certain days almost demonstrated the normal distribution, with the highest one on P600 porous scaffold. The OD values of macrophages on P600 porous scaffolds on the 3rd, 5th, and 7th day were 1.31 ± 0.08, 1.85 ± 0.12, and 2.74 ± 0.16, respectively. The OD values of macrophages on P300 and P1000 porous scaffolds were almost the lowest at all times. The OD values of macrophages on P300 porous scaffolds on the 3rd, 5th, and 7th day were 0.65 ± 0.11, 1.04 ± 0.11, and 1.28 ± 0.09, respectively. Such a result indicated that the pore size could influence cell viability and proliferation greatly.

#### 3.2.2 Immunofluorescent staining of RAW 264.7

**FIGURE 5:**
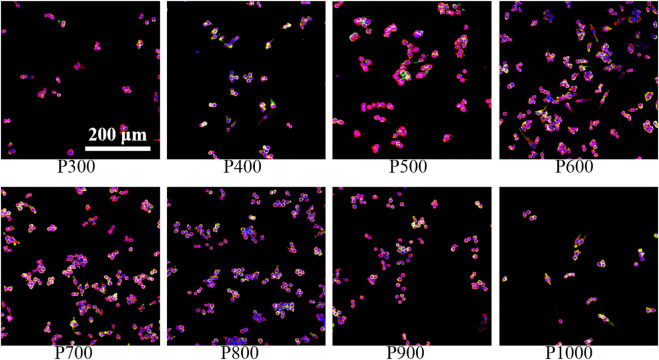
Immunofluorescence staining of macrophages on porous scaffolds with different pore sizes (red fluorescence is integrin β1, green fluorescence is cytoskeleton, blue is nucleus).

The immunofluorescence staining images on the macrophages seeded on the porous scaffolds exhibited that the expression of integrin β1 gradually enhanced with the increased pore size when the pore size was smaller than 800 μm, as shown in Figure 5. In fact, the expressions of integrin β1 in macrophages seeded on the P600, P700, and P800 porous scaffolds were much stronger. When the pore size was larger than 800 μm, the expression of integrin β1 decreased gradually.

#### 3.2.3 BMSCs osteoblastic differentiation on scaffolds

**FIGURE 6:**
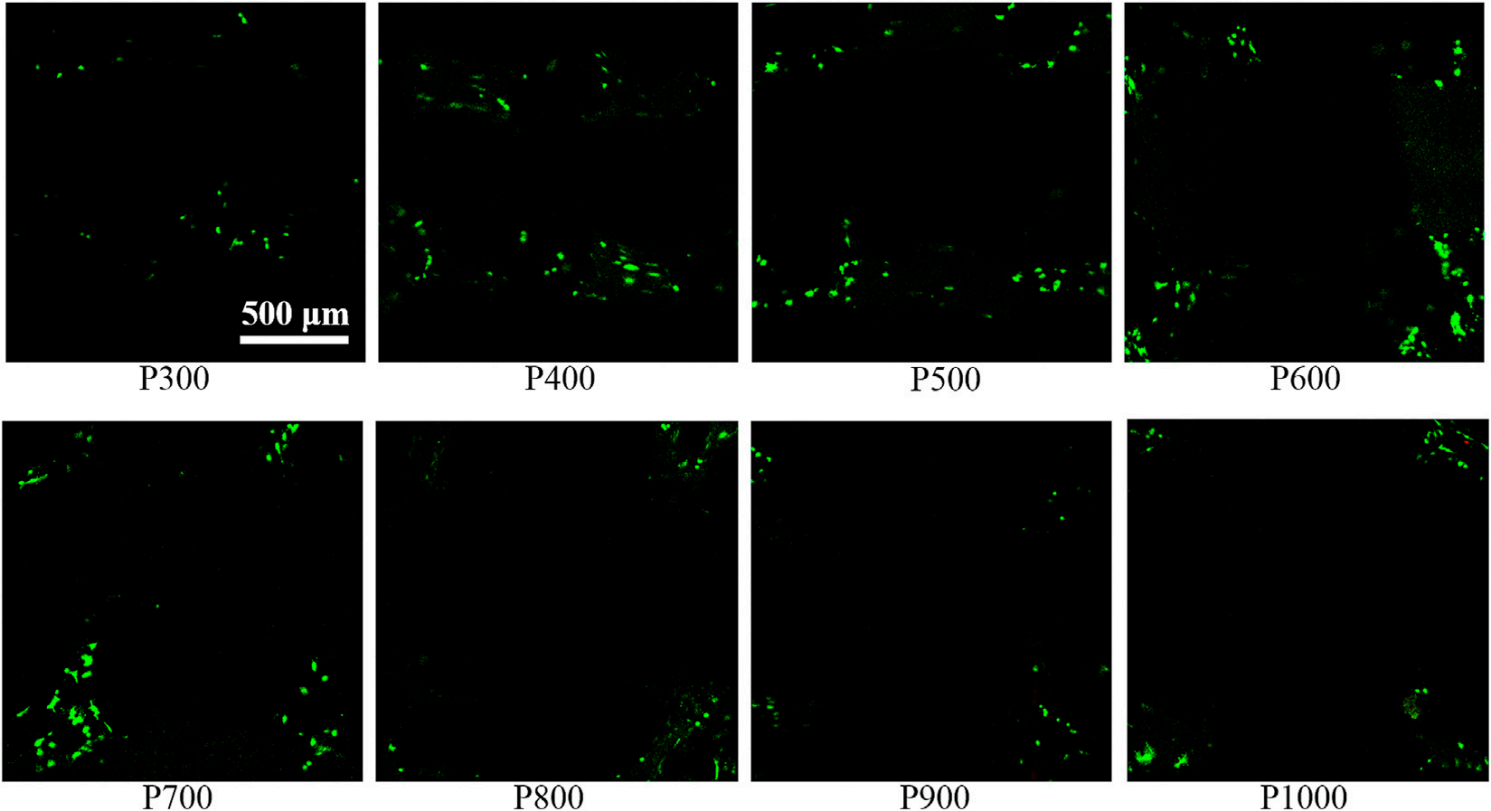
Staining image of BMSCs on porous scaffolds with different pore sizes (green fluorescent cells are living cells, red fluorescent cells are dead cells).

In order to evaluate the interaction between BMSCs and porous scaffolds with different pore sizes, the cell live/death assay, SEM observation, and osteogenesis-vascularization-related gene expression assay of BMSCs were performed. The live/dead staining image of BMSCs adhered to the porous scaffold showed that only live cells could be found, but the dead cells were almost impossible to detect, as shown in Figure 6. On the P300 scaffold, only a small number of live BMSCs were seen to adhere. With the increase of pore size, the number of live BMSCs adhered to the porous scaffold gradually increased and obtained the highest value on P600 porous scaffold. When the pore size was larger than 600 µm, the number of living BMSCs adhered to the porous scaffold gradually decreased with the increased pore size.

**FIGURE 7:**
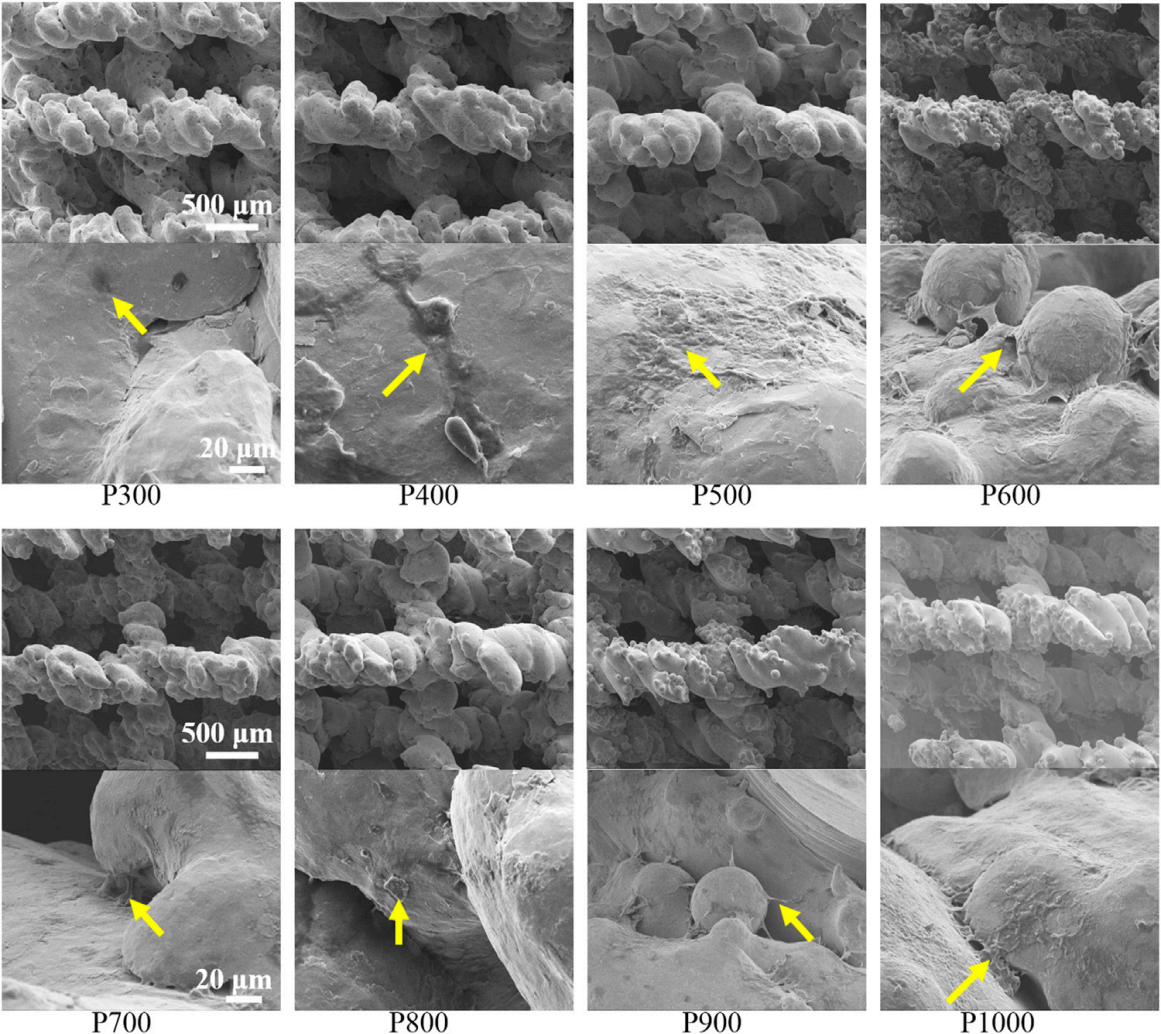
SEM images of BMSCs adhered on porous scaffolds with different pore sizes (yellow arrows point to BMSCs).

SEM observations on the BMSCs adhered on porous scaffolds with different pore sizes were shown in Figure 7. It could be found that the BMSCs preferred to adhere on the rough surfaces of porous scaffolds such as the grooves or the interfaces of unmelted alloy powders. From the SEM images with low magnification, a large number of BMSCs could be found to adhere to the porous scaffolds with different pore sizes in the form of black dots. From the SEM image with high magnification, the BMSCs protruded filamentous pseudopods that could be well observed, and some of them were aggregated into clusters. When the pore size of the porous scaffold was not larger than 600 μm, the number of adhered BMSCs increased with the increased pore size. When the pore size of the porous scaffold was larger than 600 μm, the number of adhered BMSCs decreased with the increased pore size.

#### 3.2.4 Immunofluorescent staining of BMSCs

**FIGURE 8:**
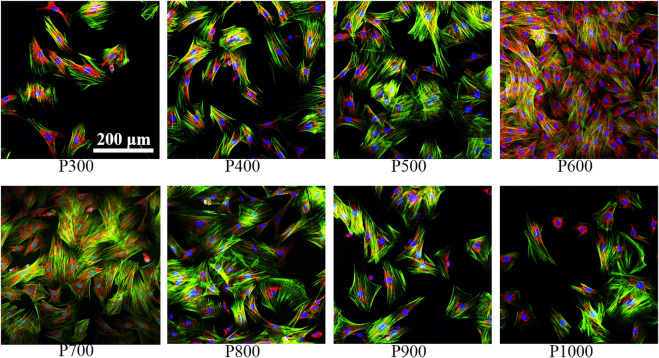
Immunofluorescence staining of BMSCs on porous scaffolds with different pore sizes (red fluorescence is integrin β1, green fluorescence is cytoskeleton, blue is nucleus).

The immunofluorescence staining images on the BMSCs adhered on different porous scaffolds were shown in Figure 8. Clearly, the number of BMSCs gradually increased with increasing pore size, and the expression of integrin β1 gradually enhanced, when the pore size was smaller than 600 μm. For the P600 porous scaffolds, the adhered BMSCs demonstrated the overlap morphology and a sheet-like distribution. When the pore size was larger than 600 μm, the expression of integrin β1 gradually decreased with the increased pore size.

**FIGURE 9:**
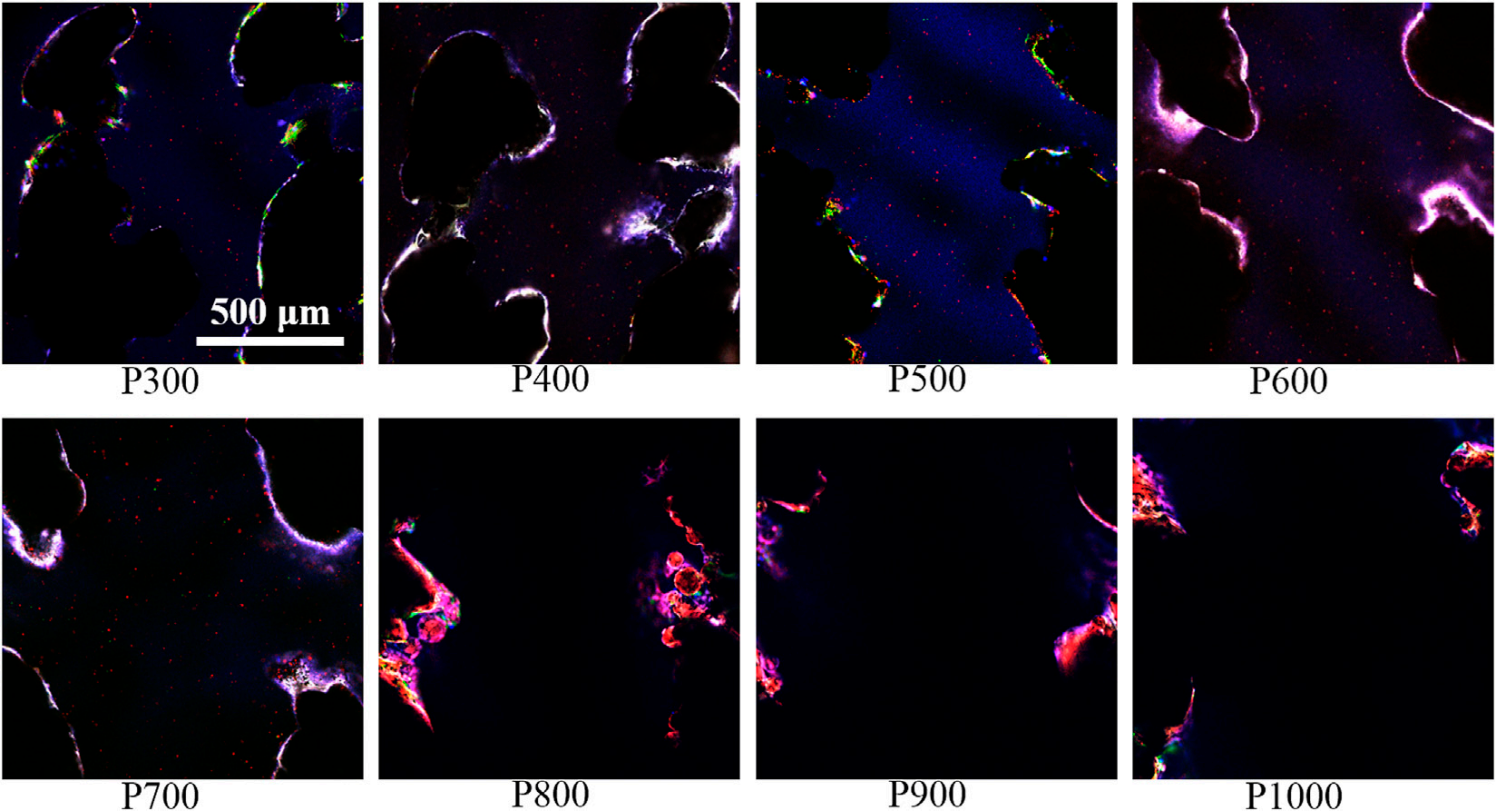
Immunofluorescence staining images of BMSCs on porous scaffolds with different pore sizes (red fluorescence is integrin β1, green fluorescence is cytoskeleton, blue is nucleus).

Further immunofluorescence staining analyses of BMSCs adhered on porous scaffolds with different pore sizes showed that they were mainly clustered at the frame edges of the scaffolds, as shown in Figure 9. Moreover, the pore contours of the scaffolds could not be fully displayed as the pore sizes of the scaffolds increased. Integrin β1 was a stress protein shown by red fluorescence, whose expression was gradually enhanced in the BMSCs adhered on the porous scaffold from pore size 300 μm–600 μm (P300 to P600). When the pore size of the porous scaffold is larger than 600 μm, the expression of integrin β1 was gradually decreased as the increased pore size. As can be seen on the P600 porous scaffold, the overlap of three colors had formed a bright white color due to the higher expression of integrin β1 (red fluorescence), cytoskeleton (green fluorescence), and nucleus (blue fluorescence), which indicated that the BMSCs had demonstrated the best activity on this kind of porous scaffolds.

#### 3.2.5 Expression of osteogenic genes related to BMSCs

**FIGURE 10:**
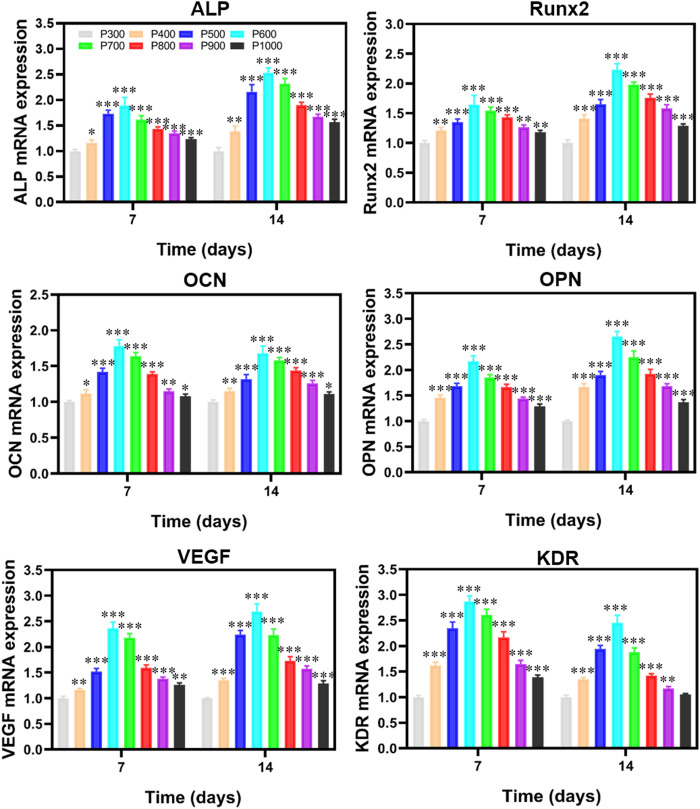
RT-PCR detection on the osteogenic gene-related expression of BMSCs cultured on porous scaffolds with different pore sizes. (*p < 0.05, **p < 0.01, and ***p < 0.001).

To further evaluate the effect of porous scaffolds with different pore sizes on the osteogenesis behavior of the BMSCs, the expression of genes associated with osteogenesis in BMSCs was tested and shown in Figure 10. It revealed that the expressions of alkaline phosphatase (ALP), Runt-related Transcription Factor 2 (Runx2), Osteocalcin (OCN), Osteopontin (OPN), vascular endothelial growth factor (VEGF) and kinase insert domain receptor (KDR) were almost exhibited the similar variations which rose with the increase of pore size and reached the maximum value at 600 μm porous scaffold, and then these expressions descended with increased pores size. Compared with the expressions of genes in BMSCs cultured on the P300 porous scaffold, those on the P500, P600, and P700 porous scaffolds were much more statistically significant. The results obtained from CCK8, cell death, and RT-PCR assays revealed that cell adhesion, proliferation, and differentiation of BMSCs cultured on the P500, P600, and P700 porous scaffolds were significantly stronger than the others. Based on the *in vitro* experimental results, the P500, P600, and P700 porous scaffolds were selected for the following *in vivo* animal experimental studies.

### 3.3 *In vivo* animal experiments

**FIGURE 11:**
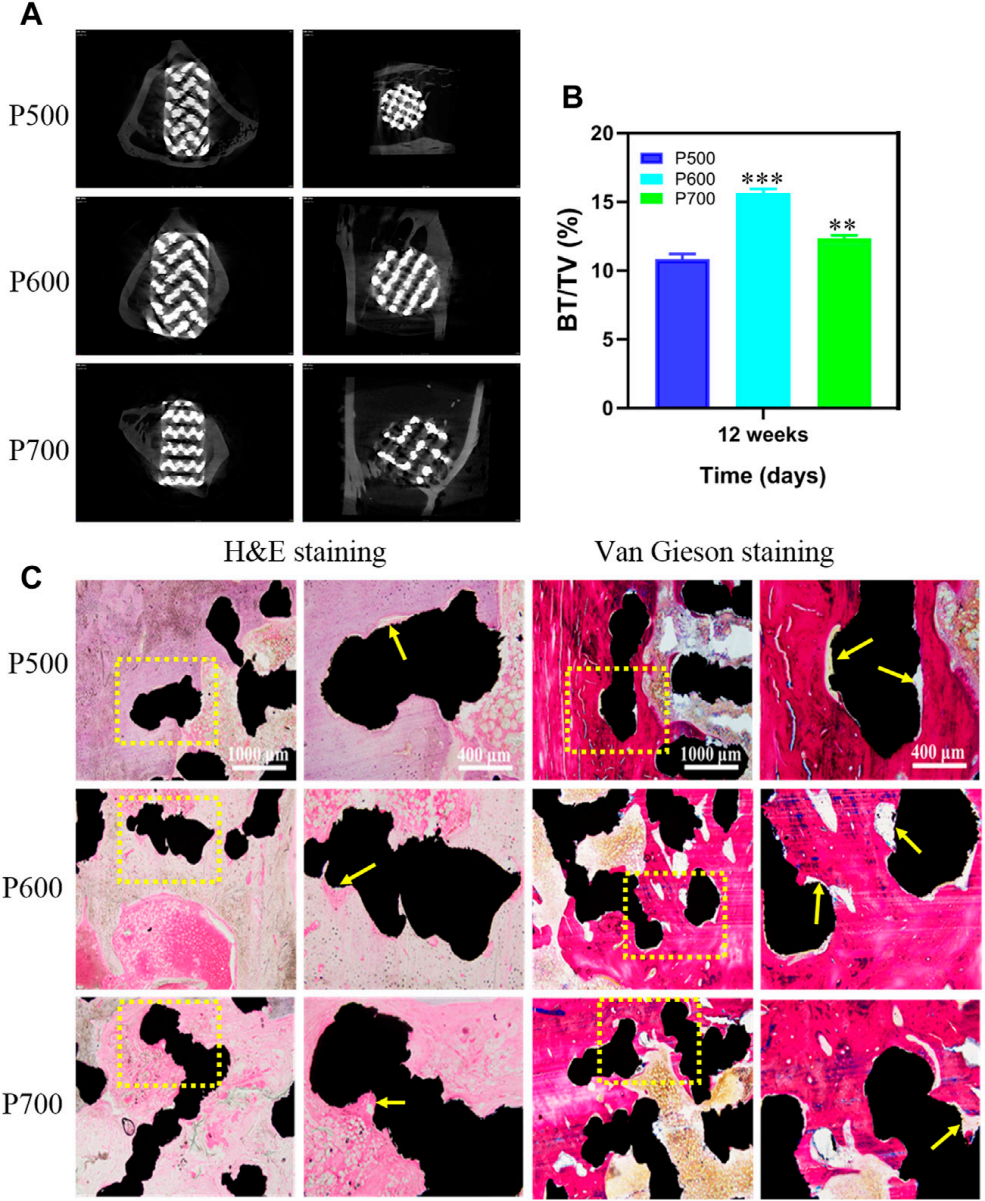
Micro-CT analysis on the porous scaffolds implanted for 12 weeks and the corresponding tissue analyses by H&E and Von Gieson staining. **(A)** Micro-CT evaluation of bone ingrowth, vertical and transverse images were presented. **(B)** Quantitative analysis of BV/TV. (*p < 0.05, **p < 0.01, and ***p < 0.001, when compared with P500.) **(C)** H&E and Von Gieson staining for detecting the new bone formation, trabecular bone ingrowth and integration in each group. (red referring to bone tissue, black referring to the porous scaffold, and the yellow arrow pointing to the new bone along the interface of scaffold and bone tissue).

To investigate the effect of pore size on bone defect repairing, the Micro-CT analyses were performed on the formal implanted with porous scaffolds and the results are exhibited in Figure 11. It could be seen that the bone tissue coverage had appeared on each scaffold after the implantation of porous scaffold for 12 weeks. The cross-sectional analyses on the porous scaffolds by Micro-CT revealed that the new bone has integrated with the P600 and P700 porous scaffolds and grew inward. In the coronal plane of Micro-CT analyses, a clear coverage of new bone tissue appeared at the top and bottom of porous scaffolds, which became much distinct in the P500 and P600 porous scaffolds (Figure 11A). Quantitative analysis of new bone formation (BV/TV) showed that after 12 weeks of the porous scaffolds were implanted into the femoral defect area, the amount of new bone formation in porous scaffolds with a pore size of 600 μm and 700 μm was significantly higher than that in porous scaffolds with a pore size of 500 μm, with statistical differences (Figure 11B).

By hard tissue slice staining, the osseointegration of implanted scaffolds was investigated further. Figure 11C depicts H&E and Von Gieson staining histological images of rabbit tibial bone defects following scaffold implantation for 12 weeks. Further tissue analyses with H&E and Von Gieso staining exhibited that the porous scaffolds with different pore sizes closely contacted with the surrounding bone tissue without any obvious inflammatory reaction, as shown in Figure 11C. The H&E and Von Gieson stained tissue showed that a large amount of new bone had formed between the porous scaffold and original bone tissue, as the yellow arrow pointed area. Compared with P500 and P700 porous scaffolds, there is the largest area of new bone tissue on the P600 porous scaffold. Moreover, the new bone tissue formed on the P600 tissue scaffold had a relatively dense bone trabecula. Based on the stained tissue analyses, it can be concluded that the regular porous scaffold with a pore size of 600 μm was more conducive to bone tissue growth and had the best osseointegration effect, which contributed to its strongest bone regeneration capacity.

## 4 Discussions

In clinic, more than 10% of patients with implant repairing have to undergo secondary surgery due to implant loosening and abscission (Sloan et al., 2018). Among these failures, osteolytic loosening plays an important role, because the following micro-motion and fretting wear would result in chronic inflammation (Berry et al., 2002). Actually, the osteolytic loosening is closely related to the interfacial stress compatibility and the high elastic modulus of conventional implants is liable to cause obvious stress concentration in specific regions and bone resorption (Chen et al., 2017; Cole et al., 2020). However, the previous research (Nune et al., 2018) (Peng et al., 2021) demonstrated that cell adhesion and proliferation could be affected obviously by the implant surface and pore size. Therefore, how to design an implant with well-compatible elastic moduli is a basic requirement to obtain excellent osseointegration or bone regeneration capability. In the present research, the porous scaffolds with different pore sizes were designed to explore the influence of pore size on the mechanical properties, osteoinduction, and osteogenesis.

### 4.1 Effect of pore size on physical and mechanical properties

The elastic modulus of the Ti6Al4V alloy is always more than 100 GPa which is much higher than that of the cortical and cancellous bone (Sen and Ramamurty, 2010). To obtain a well matching between the Ti6Al4V alloy implant and bone tissue, the porous structure is an efficient way (Li et al., 2015). However, the increased pore size and porosity not only decreases the elastic modulus but also the strength greatly. For bone defect repair, the strength of the porous implant is still important, because the porous implant could provide structural support. And moreover, the porous implant with reasonable stress action on bone tissue is beneficial to osteogenesis (Cao et al., 2019). The present research reveals that the regular cubic porous scaffold exhibits a continuous decrease in strength and elastic modulus with increased pore size. However, there is an obvious drop in strength when the elastic modulus is close to that of the cortical bone. Such a phenomenon may be ascribed to the structure stability of porous scaffolds. During the compressive deformation, the inner pore space of the porous scaffold would be consumed by the deformed frames. Due to the rapid solidification during EBM, the cubic frame has a fine grain structure, which helps to enhance the compressive strength greatly (Liu et al., 2022; Ouyang et al., 2022). However, the cubic pore structure is apt to deform, especially at larger sizes (Li et al., 2021). The deformation feature of the present porous scaffold is beneficial to the elastic modulus but detrimental to the strength. Therefore, the porous scaffold with a relatively bigger pore size has a lower elastic modulus. In addition, the frame structure in porous scaffolds with bigger pore sizes is prone to deform and fail, because of the higher stress concentration. Therefore, the regular cubic porous scaffold with a pore size of about 600 μm demonstrates the balanced strength and elastic modulus.

Since the high surface tension of the solidified Ti6Al4V alloy powder, the additive-manufactured specimens always exhibit high contact angles (Bassous et al., 2019). Though the relatively coarse surface could improve the wettability, its exerted influence is still minor. Therefore, the pore size plays an important role on the contact angle. With the increasing of pore size, the proportion of frame structure decreases and its effect is degraded gradually. Therefore, the contact angle of the Ti6Al4V alloy porous scaffold decreases with the increased pore size. The obvious drop around the pore size of 600 μm indicates that such a sized porous scaffold would have better biocompatibility, because of its benefits to the transfer of nutrition (Mohammadi et al., 2021).

### 4.2 Effect of pore size on osteoinduction and osteogenesis

Recently, many studies (Park et al., 2016; Zhang et al., 2016) have mainly focused on the proliferation and differentiation of the BMSCs affected by biomaterials directly. Few studies focus on the immunomodulatory properties of porous materials and their influence on the proliferation and differentiation of BMSCs through the immune microenvironment (Su et al., 2020). In fact, the implant would lead to an inevitable immune tissue reaction, which manifests as an acute inflammatory response during the early stage of implantation. When a porous scaffold is implanted in the bone defect area, macrophages would begin to communicate with the scaffold surface first. In the present research, the proliferation, differentiation, and OD value of RAW264.7 macrophages seeded on porous scaffolds exhibit a variation of first increasing and then decreasing with the increased pore size. Their manifestations changed when the pore size is 600 μm, which indicates the smaller or bigger pore sizes are unfavorable to the macrophages. In addition, the morphology of macrophages is usually correlated with polarization status, in which the rounded morphology is associated with a pro-inflammatory M1 phenotype and the M2 phenotype exhibits an elongated shape (McWhorter et al., 2013). The macrophages in the present research mainly exhibit the rounded morphology on all porous scaffolds, which indicates the pro-inflammatory is the main feature. Though the proliferation of macrophages has not exhibited obvious differences at the initial co-culturing stage, however, it differs greatly with the co-culturing time extending. The macrophages seeded on the porous scaffold with a pore size of 600 μm have the highest proliferation, which should be ascribed to the reasonable pore size. It is the balanced elastic modulus, strength, contact angle, and capability of nutrient transfer of the P600 porous scaffold that synergistically promotes the proliferation of macrophages (Li et al., 2018; Chen et al., 2020). Correspondingly, the integrin β1 also demonstrates the highest expression in the macrophages seeded on P600 porous scaffold, which should be attributed to the reasonable surface stress induced by the elastic modulus (Sun et al., 2019). Considering the important role of integrin β1 played in regulating macrophage differentiation and the response to external signals, it can be deduced that the porous scaffold with a pore size of about 600 μm would possess the better capability of promoting cell adhesion and proliferation (Antonov et al., 2011). Therefore, it is important to consider the role of immune cells in the osteogenesis process when evaluating the osteogenic capacity of porous scaffolds.

According to the previous research (Raz et al., 2004), the interaction between immune cells and osteoblast-related cells could play a crucial role in implant-assisted bone defect repair. Especially for the BMSCs, they act as the seed cells in bone tissue engineering and influence the whole osteogenesis processing (Guerrero et al., 2013; Qin et al., 2014). In the present research, the proliferation, integrin β1, and osteogenic gene-related expressions of BMSCs seeded on porous scaffolds with different pore sizes all exhibit similar variations which increase with increased pore size firstly, reaching the maximum value at pore size about 600 μm, and then decrease subsequently. Moreover, such variations of BMSCs are strengthened with the co-culturing time, which indicates the activity of cells on the scaffolds is closely related to the porosity. Such a finding is consistent with the previous investigations, which reveal a negative relationship between pore size and cell adhesion after its colonization on scaffolds for 24 h because the increased local stress concentration, fluid velocity, and vortex formation with increased pore size affects the cell colonization (Torres-Sanchez et al., 2021). The variation of integrin β1 also demonstrates the influence of surface morphology, which works by regulating the plasma membrane receptor integrin with extracellularly adsorbed interacting matrix proteins (Degasne et al., 1999). In the present research, the porous scaffolds with smaller pore sizes have more coarse surfaces, but the unmelted powders may exert negative effects, while the porous scaffolds with bigger pore sizes have less coarse surfaces due to the decreased frame amount. Such a surface feature evolution of porous scaffold influences the protein adsorption differently, which results in the highest integrin β1 expression of BMSCs seeded on porous scaffolds with a pore size of 600 μm. A previous study showed that osteoblasts growing on a slightly rough surface could produce an osteogenic environment that promoted osteoblast differentiation through paracrine and autocrine pathways (Boyan et al., 2003).

The expression of ALP in the formation and maturation of extracellular bone matrix, the expression of OCN and OPN in the mineralization of bone matrix, and the promotion of bone tissue mineralization by Runx2 are the main markers of osteogenic differentiation (Chang et al., 2016; Ou and Huang, 2021). VEGF plays an important role in almost all important stages of angiogenesis by inducing endothelial cell migration, proliferation, and tubular formation (Lamalice et al., 2007). It has been reported that VEGF can induce angiogenesis by interacting with KDR, a tyrosinase (Hoppe et al., 2011). All these osteogenic gene-related expressions of BMSCs lose their increasing tendency when the pore size of the porous scaffold is larger than 600 μm, which may be ascribed to the delayed differentiation process of the initial stage of osteogenesis by excessive pore size (Chang et al., 2016). It is worth noting the osteogenic and angiogenic genes expressions of BMSCs co-cultured and seeded on P600 porous scaffold were significantly higher than others, which may be explained by the mechanical transmission mechanism (Murphy et al., 2010; Van Bael et al., 2012). The *in vivo* experiment confirms the *in vitro* results, which further verifies the deduction the wettability, permeability, elastic modulus, and strength synergistically influence the osteoinduction and osteogenesis behavior of the porous scaffold (Houdek et al., 2020; Akram et al., 2021). The porous scaffolds with a pore size of 600 μm possess the better capability to induce new bone formation, due to their well-balanced physical and mechanical properties. Therefore, for the design of a Ti6Al4V alloy regular porous scaffold, the surface morphology, porosity, strength, and elastic modulus should be the main considering factors, which determine its capability of osteoinduction and osteogenesis.

## 5 Conclusion

The Ti6Al4V alloy porous scaffolds with gradient pore sizes were designed and fabricated to systematically evaluate the effect of pore size on mechanical properties, osteoinduction, and osteogenesis. Based on the experimental results, the following conclusion could be drawn.

(1) With the increasing pore size, the contact angle with water, compressive strength, and elastic modulus of the Ti6Al4V alloy porous scaffold decreased gradually. However, these properties had obvious drops when the pore size of porous scaffolds located around 600 μm.
(2) As the pore size increased, the proliferation and integrin β1 of RAW 264.7 macrophages seeded on Ti6Al4V alloy porous scaffolds increased firstly, reaching a maximum value at a pore size of about 600 μm, and then decreased subsequently.
(3) The proliferation, integrin β1, and osteogenic gene-related expressions of BMSCs seeded on Ti6Al4V alloy porous scaffolds with different pore sizes all exhibited similar variations which increased with increased pore size firstly, obtaining the maximum value at pore size about 600 μm, and then decreased.
(4) The *in vivo* experiment confirmed the *in vitro* results, and the Ti6Al4V alloy porous scaffolds with a pore size of 600 μm possessed the better capability to induce new bone formation. Therefore, for the design of a Ti6Al4V alloy regular porous scaffold, the surface morphology, porosity, strength, and elastic modulus should be considered systematically, which would determine the capability of osteoinduction and osteogenesis.

## Data Availability

The raw data supporting the conclusion of this article will be made available by the authors, without undue reservation.
